# Apixaban in Comparison to Warfarin for Stroke Prevention in Nonvalvular Atrial Fibrillation: A Systematic Review and Meta-Analysis of Observational Studies

**DOI:** 10.1155/2019/6419147

**Published:** 2019-07-07

**Authors:** Muhammad U. Siddiqui, David Scalzitti, Zunaira Naeem

**Affiliations:** ^1^Department of Medicine, Marshfield Clinic, Rice Lake, WI 54701, USA; ^2^Department of Biostatistics, George Washington University, Washington, DC 20052, USA; ^3^Department of Medicine, Marshfield Clinic, Chippewa Falls, WI 54721, USA

## Abstract

**Introduction:**

Atrial fibrillation leads to increased risk of systemic embolism and stroke. To decrease these adverse events, anticoagulation is routinely prescribed. Nonvitamin K anticoagulants like apixaban and rivaroxaban are becoming popular and being used more frequently nowadays. We here compare the efficacy and safety of apixaban with those of warfarin.

**Methods and Analysis:**

This systematic review aims to assess the efficacy and safety of apixaban compared to those of warfarin. Eligible participants were adults diagnosed with nonvalvular atrial fibrillation. The intervention was apixaban, and the comparator was warfarin. The primary efficacy endpoint is the first admission with systemic embolism or stroke, and the primary safety outcome is the occurrence of major bleeding. Relevant studies were searched in the Cochrane Central Register of Controlled Trials, MEDLINE, PubMed, and clinicaltrials.gov. After being independently reviewed by two authors, five articles were included in the systematic review. The risk of bias of included studies was assessed using the Cochrane risk of bias tool and SIGN methodology. The RevMan software was used to assess the effect size and perform meta-analysis.

**Results:**

Apixaban was found to be superior to warfarin in terms of safety (RR 0.58; CI 0.52–0.66) but not superior to warfarin in terms of efficacy (RR 0.93; CI 0.70–1.24).

**Conclusion:**

Apixaban is superior to warfarin in terms of safety, but no difference in efficacy is noted. The choice of anticoagulation should be individualized based on the risk factor profile of the patient.

## 1. Introduction

### 1.1. Background

Atrial fibrillation (AF) is an irregular heart rhythm. It is considered the most common cardiac arrhythmia and increases the risk of strokes by fivefold [1]. In order to prevent strokes, anticoagulants are routinely prescribed, particularly in population with the CHA_2_DS_2_-VASc score greater than or equal to 2 [2]. CHA_2_DS_2_-VASc and CHADS_2_ risk models are preferred tools to estimate the risk of embolic strokes in patients with atrial fibrillation [3, 4]. The CHA_2_DS_2_-VASc tool assigns a score of 0, 1, or >2 to the individuals with atrial fibrillation. The benefits of anticoagulation significantly outweigh the risk in almost all patients with nonvalvular atrial fibrillation with a CHA_2_DS_2_-VASc score equal to or greater than 2 [2, 5]. Warfarin reduces the risk of strokes by 68% and however requires regular international normalized ratio testing and has frequent interactions with multiple drugs and food. In recent years, researchers have overcome these shortcomings by introducing a new class of anticoagulants called nonvitamin K oral anticoagulants (NOACs). Clinical trials have demonstrated that NOACs are equivalent to warfarin in effectiveness and safety and therefore are now routinely used in practice [6–9].

Among the NOACs, there are factor Xa inhibitors (apixaban, rivaroxaban, and edoxaban). A 2014 Cochrane review compared factor Xa inhibitors to warfarin and identified lower rates of strokes and bleeding with factor Xa inhibitors [10]. This review did not compare the efficacy and safety of different agents among factor Xa inhibitors with each other. To answer this question, head-to-head trials are required which are lacking. Hence, investigators have tried to answer this question by performing network analysis and indirectly comparing these agents [11, 12]. These trials found similar effectiveness in preventing strokes but found lower risk of major bleeding with apixaban. Due to lack of direct comparison, these results are not fully incorporated in clinical practice and the decision is left up to the clinician's preference.

As NOACs are becoming popular and more frequently used, new trials and evidence are constantly being reported. A systematic review of observational studies recently reported a low risk of stroke/systemic embolism and major bleeding with apixaban when compared to warfarin [13]. In order to further scrutinize and incorporate this new evidence in clinical practice, we decided to perform a combined systematic review of randomized and nonrandomized controlled trials to find if both regular-dose apixaban (5 mg) and reduced-dose apixaban (2.5 mg) are superior/noninferior to warfarin in terms of efficacy and safety.

### 1.2. Objectives

The objectives of this study are to determine efficacy and safety of apixaban in comparison to those of warfarin for preventing strokes in patients of 18 years old and above with nonvalvular atrial fibrillation.

## 2. Methods

### 2.1. Eligibility Criteria

This review included randomized controlled trials (RCTs) and non-RCTs (case-control and cohort) comparing efficacy and safety of apixaban vs those of warfarin in patients with nonvalvular atrial fibrillation. Studies with participants 18 years or older with nonvalvular atrial fibrillation using apixaban or warfarin to prevent stroke were included (Table S1). Inclusion criteria included a minimum 6-month continuous follow-up after initiation of apixaban or warfarin. Patients were also required to have AF diagnosis (International Classification of Disease, 9^th^ Revision, diagnosis code 427.1) at baseline. Patients on dialysis and having valvular atrial fibrillation were excluded from the study.

The first admission with stroke or systemic embolism was considered the primary efficacy outcome. The first admission with major bleeding (gastrointestinal bleeding, intracranial bleeding, and bleeding from other sites) was the primary safety outcome.

### 2.2. Search Strategy

We performed a systemic search on February 7, 2018, on the following databases to search for relevant articles:

1. MEDLINE (Ovid)
2. Cochrane Central Register of Controlled Trials
3. PubMed
4. Clinicaltrials.gov

The keywords that were used to perform searches included atrial fibrillation AND stroke prevention/control AND apixaban OR factor Xa inhibitors OR pyridines AND warfarin. The keywords were inserted in the medical term (MeSH) search bar, available in the MEDLINE and Cochrane registry. They were then combined using AND/OR, as described earlier. To search for grey literatures and unpublished articles, we used the above-mentioned keywords to perform search in clinicaltrials.gov. Once search was performed, the articles were exported to legacy RefWorks. A search for duplicates was performed in RefWorks, and duplicate articles were removed.

### 2.3. Data Collection and Analysis

Two review authors (MUS and ZN) independently reviewed full text of the articles selected for eligibility. If there were any disagreements, a third review author (DS) was asked to arbitrate. A data collection form was created on Microsoft Word and was shared with the second author. The data regarding study characteristics, intervention, comparison, and outcomes were documented on the form (Tables S1–S3). Two authors independently extracted outcome data on a standardized data extraction tool. Any disagreements were resolved by discussion. One author transferred data into Review Manager (RevMan).

### 2.4. Assessment of Risk of Bias

Risk of bias for each study was assessed by two authors independently using the criteria outlined in the Cochrane Handbook for Systematic Reviews of Interventions. Any disagreements were resolved by discussion. For RCTs, the Cochrane risk of bias tool for RCTs was utilized (Table S4). For cohort studies, the SIGN methodology was utilized to assess the risk of bias (Table S5). Each potential source of bias was graded as high, low, or moderate.

### 2.5. Data Synthesis

The statistical analysis was undertaken using Review Manager (RevMan) version 5.3 (The Cochrane Collaboration 2014, Nordic Cochrane Centre, Copenhagen, Denmark). Outcomes from each study were pooled and compared using a random-effects model according to the heterogeneity between all included studies. The treatment effect was reported as risk ratio (RR) and 95% confidence interval (CI). The *I* ^2^ statistic was quantified to measure heterogeneity, and the Mantel–Haenszel (M-H) random-effects model was used. A *p* value <0.05 was considered statistically significant.

## 3. Results

### 3.1. Study Selection

**Figure 1:**
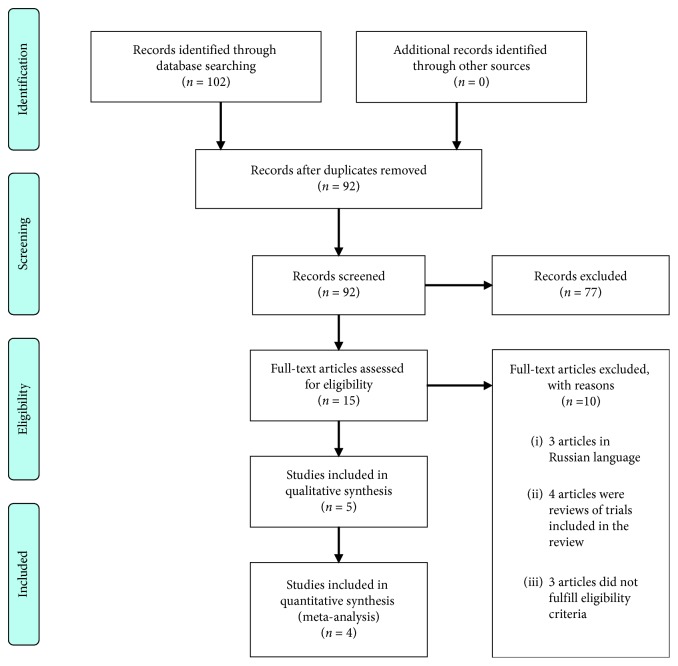
PRISMA flow diagram.

One hundred two potential articles were found after search (Figure 1). One author independently screened titles and abstracts of the articles. A PRISMA flow diagram (Figure 1) is included for details. The full texts of 15 articles were retrieved for assessment of eligibility. 10 articles were excluded after the full-text review. Among excluded articles, 3 were found to be in Russian language, 4 articles were reviews of studies already included in the meta-analysis, and the other excluded articles did not fulfill the inclusion criteria. 5 articles were selected to perform the systematic review [8, 14–17]. The second author (ZN) reviewed these five articles independently to assess eligibility. If there were any disagreements, the third review author (DS) was asked to arbitrate.

### 3.2. Study Characteristics

Overall, 65199 patients were treated with apixaban in the included five studies. Four among five studies were observational cohort studies [14–17], while one study was a randomized controlled trial [8]. The one randomized controlled trial was published in 2011 [8], and the four observational cohort studies were published between 2016 and 2018 [14–17]. Two studies were conducted in the United States [8, 15], whereas three studies were conducted in Europe [14, 16, 17]. The baseline characteristics were consistent in all the studies. Three studies reported age data using median and interquartile range (IQR) [8, 14, 17]. Since these three studies had large sample size, they were considered symmetrically distributed. Therefore, the median was estimated to be equal to mean, and the IQR was divided by 1.35 to estimate standard deviation. The mean age of patients taking apixaban ranged between 68.6 and 71.7 in three studies [8, 14, 15]. Two studies separately reported patients taking reduced-dose apixaban [15, 16]. Patients on reduced-dose apixaban are usually older with more comorbidities. Hence, in these two studies, the mean age of patients on reduced-dose apixaban ranged between 82.5 and 83.5. One study included patients taking both low- and high-dose apixaban but did not perform a subgroup analysis in terms of the dose being utilized [17]. The mean age of the apixaban group in this study was 76.

### 3.3. Risk of Bias Evaluation

**Figure 2:**
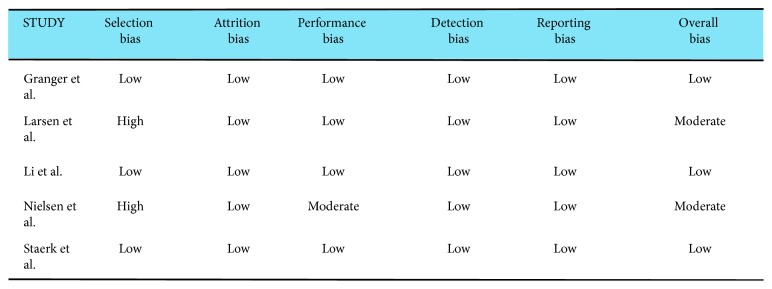
Risk of bias.

A risk of bias evaluation was performed using the Cochrane risk of bias tool (Table S4) for randomized trials [8] and the SIGN tool (Table S5) for observational cohort studies [14–17]. All five studies overall reported a low risk of bias (Figure 2). We did not find significant publication bias in the main primary outcome, for comparison between apixaban and warfarin.

### 3.4. Study Results

Risk ratio was used as a summary measure. In two studies [8, 15] comparing apixaban with warfarin, apixaban was associated with a lower risk of stroke and systemic embolism. Three studies [14, 16, 17] showed no difference in the risk of stroke and systemic embolism.

Apixaban was associated with a lower risk of major bleeding compared to warfarin in three studies [8, 14, 15]. One study [16] did not show any difference in the risk of major bleeding.

### 3.5. Meta-Analysis of Selected Studies

We only analyzed the four observational studies for the meta-analysis [14–17] and excluded the trial conducted by Granger et al. since it was a randomized trial. We compared the meta-analysis results with the findings from the only randomized trial [8] for similarities/differences.

#### 3.5.1. Efficacy Outcome

**Figure 3:**
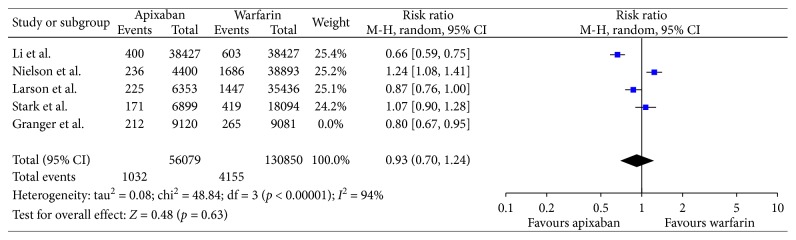
Efficacy with only nonrandomized trials.

In our analysis of nonrandomized trials, there was no difference in reducing stroke and/or systemic embolism when comparing apixaban with Coumadin (risk ratio, 0.93; 95% confidence interval (95% CI), 0.70–1.24; Figure 3). Analysis can be interpreted as clinically favoring apixaban over warfarin, and however, as mentioned earlier, the results were not statistically significant.

The result was slightly different from that of the randomized trial of Granger et al. which similarly to our meta-analysis found that apixaban is better in reducing strokes/SE in relation to warfarin. However, in contrast to our meta-analysis, the result was statistically significant.

#### 3.5.2. Safety Outcome

**Figure 4:**
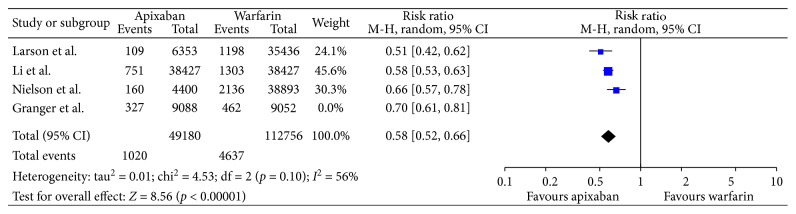
Safety with only nonrandomized trials.

Analysis of nonrandomized trials for major bleeding identified a statistically significant difference favoring apixaban when compared to warfarin (risk ratio, 0.58; 95% confidence interval (95% CI), 0.52–0.66; Figure 4).

The result was similar to that of the randomized trial by Granger et al. When it comes to major bleeding, a significant difference was noted favoring apixaban over warfarin in the trial performed by Granger et al.

#### 3.5.3. Heterogeneity

An increased level of statistical heterogeneity was noted in the meta-analysis for both the outcomes. Lack of randomization in the four observational studies [14–17] is one possible reason for heterogeneity. Two included trials separately reported the comparison between reduced-dose apixaban (2.5 mg) and warfarin [15, 16]. Reduced-dose apixaban is used in older population with age >80 years, and therefore, the mean age of study population in the reduced-dose apixaban trials was 82.5–83.5. It is well known that the risk of stroke, death, and major bleeding increases with increased age [18]. Therefore, the increased mean age in the reduced-dose apixaban trials also contributed to the statistical heterogeneity.

## 4. Discussion

We reviewed five studies to determine efficacy of apixaban compared to that of warfarin and reviewed four studies to compare the safety profile of apixaban vs that of warfarin. However, the meta-analysis was performed using only observational studies [14–17]. The total number of patients who have their bleeding and thromboembolic risk assessed was >49000. The study denotes that patients who were taking warfarin had more bleeding events when compared to those who were on apixaban, and the result was statistically significant. However, there was no statistical difference between thromboembolic events in both arms. Previous meta-analysis performed by Proietti et al. favored apixaban over warfarin both in terms of efficacy and safety. However, the results for efficacy were statistically nonsignificant [13] similar to our conclusion. They further performed subgroup analysis and found that, in the reduced-dose apixaban group, the reduction in thromboembolic diseases was statistically significant. Proietti et al. did not include the phase III ARISTOTLE trial [8] comparing apixaban and warfarin in contrast to our systemic review. The ARISTOTLE landmark trial found that apixaban had statistically significant reduction in stroke/systemic embolism as well as major bleeding when compared to warfarin.

Out trial further reinforces the superiority of apixaban over warfarin. To date, there is only one randomized controlled trial on this topic [8]. Several nonrandomized cohort studies have however been reported. Though there is paucity of randomized trials, the trials so far performed have clearly shown clinical benefits of apixaban over warfarin. Other NOACs have shown similar superiority over warfarin. Therefore, NOACs are now being used more frequently in the real-world setting and being preferred over warfarin [13, 19]. Given mounting evidence, the guidelines have changed in favor of NOACs over warfarin [20].

Though the result of this meta-analysis is consistent with that of similar previous studies, an individualized approach should be exercised in managing each patient. This meta-analysis did not include patients who had valvular atrial fibrillation or patients who were on dialysis, and therefore, the results from this study cannot be applied on them. Having said that, Granger et al. performed subgroup analysis and found that apixaban was safer and caused less major bleeding episodes in patients with severe renal impairment when compared to warfarin. Apixaban was also found to have similar efficacy but a better safety profile in patients with diabetes mellitus [8]. Our meta-analysis had two trials that compared reduced-dose apixaban with warfarin [15, 16]. As described in Methods, reduced-dose apixaban is used in elderly population or patients with kidney disease [8]. Nielsen et al. compared reduced-dose apixaban with warfarin and found more thromboembolic and major bleeding events with apixaban. The ORBIT-AF II trial (Outcome Registry for Better Informed Treatment of Atrial Fibrillation Phase II trial) showed that 11.8% of patients with atrial fibrillation are undertreated with reduced-dose apixaban, and this leads to worse outcomes [21].

The four nonrandomized observational studies in our meta-analysis did not provide data on international normalized ratio (INR) values for the patients taking warfarin as this information was not available in the dataset. Granger et al. reported that the INR was in the therapeutic range (2.0 to 3.0) in the warfarin group for a mean of 62.2% of the time. Since the therapeutic window of warfarin is narrow, clinicians and researchers may argue that the favorable profile of apixaban when compared to warfarin may be due to poor quality of INR control. However, Wallentin et al. found apixaban to be more efficacious with a better safety profile than warfarin regardless of the centre's or patient's quality of INR control [22].

Overall, there was a low risk of bias associated with all the included studies (Figure 2). Given four out of the five included trials were nonrandomized studies, there is always a risk of selection bias. Two trials were noted to have a high selection bias [14, 16]. One trial was noted to have a moderate risk of performance bias [16]. Future randomized trials may be able to amend these drawbacks. All the trials reported a low risk of attrition, detection, and reporting biases. The one randomized trial included in our meta-analysis did not describe the method utilized for random sequence generation mildly affecting the quality of evidence [8].

Compared to previous meta-analysis, we have included the one randomized trial published to date in our systematic review. The outcomes however have remained the same. Similar to previous meta-analysis, regular-dose apixaban is found to have similar efficacy but better safety compared to warfarin. The effectiveness of reduced-dose apixaban is still debatable and needs further research. Our review agrees with previous similar reviews and further reinforces the favorable profile of apixaban when compared to warfarin.

## 5. Limitations

There are a few limitations in our review: First, we were unable to verify participant compliance with assigned medication. Second, there are lack of randomized trials and better quality of evidence. The included studies had increased heterogeneity, confirmed by large *I* ^2^ values. Finally, due to lack of resources, we were unable to perform the search in EMBASE. Hence, it is possible that there are other studies matching our inclusion criteria that are not included in our meta-analysis.

## 6. Conclusion

In this systematic review and meta-analysis, apixaban was associated with a better safety profile when compared to warfarin with less major bleeding events. The analysis also favored apixaban in terms of reducing strokes/systemic embolism, and however, this result was not statistically significant. Our analysis included the only randomized trial performed on this topic, and the results are consistent with those of previous systematic reviews. This review further reinforces the superiority of apixaban in comparison to warfarin in patients with nonvalvular atrial fibrillation.

## Data Availability

The data used to support the findings of this study and review protocol are available from the corresponding author upon request.

## Supplementary Materials

Supplementary Materials Supplementary Table 1: PICO characteristics of the studies included in the systematic review and meta-analysis. Supplementary Table 2: baseline characteristics of the studies included in the systematic review and meta-analysis. Supplementary Table 3: outcomes of the studies included in the systematic review and meta-analysis. Supplementary Table 4: Cochrane risk of bias tool for randomized controlled trials. Supplementary Table 5: SIGN risk of bias tool for nonrandomized controlled trials.
