# Supplementary material for: Apixaban in Comparison to Warfarin for Stroke Prevention in Nonvalvular Atrial Fibrillation: A Systematic Review and Meta-Analysis of Observational Studies
Source: Cardiol Res Pract. 2019 Jul 7;2019:6419147. doi: 10.1155/2019/6419147 (PMC6642773; doi:10.1155/2019/6419147)
Supplement: Supplementary Materials — Supplementary Table 1: PICO characteristics of the studies included in the systematic review and meta-analysis. Supplementary Table 2: baseline characteristics of the studies included in the systematic review and meta-analysis. Supplementary Table 3: outcomes of the studies included in the systematic review and meta-analysis. Supplementary Table 4: Cochrane risk of bias tool for randomized controlled trials. Supplementary Table 5: SIGN risk of bias tool for nonrandomized controlled trials. [file 6419147.f1.pdf]

Supplementary Material:

APIXABAN VS WARFARN INTERVENTION						
STUDY	POPULATION	INTERVENTION	COMPARISON	OUTCOMES	DURATION	STUDY DESIGN
Granger, CB et al.	Adults with age of at least 80 years, a body weight of no more than 60 kg, or a serum creatinine level of 1.5 mg per deciliter (133 µmol per liter) or more.	Apixaban or matching placebo was administered twice daily, with apixaban given in 5-mg doses; 2.5-mg doses were used in a subset of patients	Warfarin (or matching placebo) was provided as 2-mg tablets and was adjusted to achieve a target international normalized ratio (INR) of 2.0 to 3.0.	Apixaban was associated with lower rate of ischemic stroke/systemic Embolism and bleeding.	From December 19, 2006, through April 2, 2010.	Randomized controlled trail.
Larsen, TB et al.	Adults with non-valvular atrial fibrillation who were naive to oral anticoagulants and had no previous indication for valvular atrial fibrillation or venous thromboembolism.	Apixaban 5mg twice daily.	Warfarin 2.5mg tablets with target international normalized ratio (INR) of 2.0 to 3.0.	Apixaban was associated with lower rate of ischemic stroke/systemic Embolism and bleeding.	From December 10, 2012 through November 30, 2015.	Observational cohort study.
Li, X et al.	Adults (aged 18 years) with nonvalvular atrial fibrillation who had a pharmacy claim for apixaban or warfarin during the identification period.	Apixaban 5mg twice daily and 2.5 mg bid.	Warfarin with target international normalized ratio (INR) of 2.0 to 3.0.	Apixaban was associated with lower rate of ischemic stroke/systemic Embolism and bleeding.	From January 1, 2012 through September 30, 2015.	Observational cohort study.
Nielsen, PB et al.	Adults with non-valvular atrial fibrillation with a first prescription for an oral anticoagulant.	Apixaban 2.5mg bid were identified and followed.	Warfarin with target international normalized ratio (INR) of 2.0 to 3.0.	Apixaban with elevated risk of ischemic stroke/systemic embolism and major bleeding.	From December 10, 2012 through February 28, 2016.	Observational cohort study.
Staerk, L et al.	Adults with non-valvular atrial	Apixaban 5mg twice daily and 2.5 mg bid.	Information regarding international	Apixaban with elevated risk of ischemic	From August 22, 2011 through	Observational cohort study.

	fibrillation with a first prescription for an oral anticoagulant.		normalized ratio (INR) not available.	stroke/systemic embolism.	December 31, 2015.	

Table 1: PICO characteristics of the studies.

		Gender			Age			
Reference	Group	M	F	Both	Mean	SD	Median	Range
Granger, CB et al.	Apixaban	5886	3234	9120			70	63 - 76
	Warfarin	5899	3182	9081			70	63 - 76
Larsen, TB Et al.	Apixaban	3831	2522	6353			71.73	65.8 – 77.2
	Warfarin	20838	14598	35436			72.4	64.7 – 79.8
Li, X et al	Apixaban(5mg)<br>Apixaban(2.5mg)	20,007<br>2756	11820<br>2760	31827<br>6600	68.6<br>82.5	11.0<br>9.5		
	Warfarin*<br>Warfarin**	20.048<br>3844	11779<br>3840	31827<br>6600	69.2<br>80.1	11.7<br>8.5		
Nielsen, PB er al.	Apixaban	1735	2665	4400	83.9	8.2		
	Warfarin	23190	15703	38893	71	12.6		
Staerk, L et al.	Apixaban	3439	3460	6899			76	68 - 84
	Warfarin	10265	7829	18094			73	65 - 80

Table 2: Baseline characteristics from each study. *Baseline characteristics propensity matched to apixaban 5mg. ** Baseline characteristics propensity matched to apixaban 2.5mg.

REFERENCE/OUTCOMES		APIXABAN		WARFARIN			
		N(n)	Event rate	N(n)	Event rate	HR(CI)	P value
Granger, CB et al.	Stroke/systemic embolism	9120 (212)	1.27	9081 (265)	1.60	0.79 (0.66, 0.95)	0.01
	Major bleeding	9088 (327)	2.13	9052 (462)	3.09	0.69 (0.60, 0.80)	<0.001
Larsen, TB et al.	Stroke/systemic embolism	6353 (225)	3.32	35436 (1447)	2.33	1.08 (0.91, 1.27)	
	Major bleeding	6353 (109)	2.15	35436 (1198)	2.98	0.61 (0.49, 0.75)	
Li, X et al.	Stroke/systemic embolism	31827 (299)*	2.15	31827 (440)	3.04	0.70 (0.60, 0.81)	<0.001
		6600 (101)**	3.51	6600 (163)	5.28	0.63 (0.49, 0.81)	<0.001
	Major bleeding	31827* (563)	4.05	31827 (977)	6.80	0.59 (0.53, 0.66)	<0.001
		6600 (188)**	6.56	6600 (326)	10.64	0.59 (0.49, 0.71)	<0.001
Nielsen, PB et al.	Stroke/systemic embolism	4400 (236)	3.98	38893 (1686)	2.68	1.19 (0.95, 1.49)	
	Major bleeding	4400 (160)	3.90	38893 (2136)	3.14	1.04 (0.76, 1.43)	
Staerk, L et al.	Stroke/systemic embolism	6899 (171)		18094 (419)		1.07 (0.87, 1.31)	

Table 3: Study results. *Apixaban 5mg vs warfarin. **Apixaban 2.5mg vs warfarin.

REFERENCE	SEQUENCE GENERATION	ALLOCATION CONCEALMENT	BLINDING PARTICIPANTS	BLINDING ASSESSORS	INCOMPLETE OUTCOME DATA	SELECTIVE REPORTING	OTHER BIAS
Granger, CB et al.	Yes	Yes	Yes	Yes	No	No	None

Table 4: Cochrane risk of bias tool for randomized controlled trials.

QUESTIONS	Larsen, TB et al.	Li, X et al.	Nielsen, PB et al.	Staerk, L et al.
The study addresses an appropriate and clearly focused question.	Yes	Yes	Yes	Yes
The two groups being studied are selected from source populations that are comparable in all respects other than the factor under investigation.	Yes	Yes	Yes	Yes

The study indicates how many of the people asked to take part did so, in each of the groups being studied.	No	No	No	No
The likelihood that some eligible subjects might have the outcome at the time of enrolment is assessed and taken into account in the analysis.	Yes	Yes	Yes	Yes
What percentage of individuals or clusters recruited into each arm of the study dropped out before the study was completed.	NA	NA	NA	NA
Comparison is made between full participants and those lost to follow up, by exposure status.	NA	NA	NA	NA
The outcomes are clearly defined.	Yes	Yes	Yes	Yes
The assessment of outcome is made blind to exposure status. If the study is retrospective this may not be applicable.	NA	NA	NA	NA
Where blinding was not possible, there is some recognition that knowledge of exposure status could have influenced the assessment of outcome.	Yes	Yes	NA	Yes
The method of assessment of exposure is reliable.	No	No	No	No
Evidence from other sources is used to demonstrate that the method of outcome assessment is valid and reliable.	Yes	Yes	Yes	Yes
Exposure level or prognostic factor is assessed more than once.	No	No	No	No
The main potential confounders are identified and taken into account in the design and analysis.	Yes	Yes	Yes	Yes
Have confidence intervals been provided?	Yes	Yes	Yes	Yes

Table 5: SIGN risk of bias tool for non-randomized controlled trials.
